# An Unusual Presentation of Papillary Thyroid Carcinoma That Resembles Lymphoma: A Case Report

**DOI:** 10.7759/cureus.72381

**Published:** 2024-10-25

**Authors:** Walter Lim Yung Chwen, Azwan Halim Abdul Wahab, Khairunisa Ahmad Affandi

**Affiliations:** 1 Otolaryngology - Head and Neck Surgery, International Islamic University Malaysia, Pahang, MYS; 2 Otolaryngology - Head and Neck Surgery, International Islamic University Malaysia, Kuantan, MYS; 3 Pathology, International Islamic University Malaysia, Kuantan, MYS

**Keywords:** cervical lymphadenopathy, lateral neck swelling, lymphoma, metastasis, papillary thyroid carcinoma

## Abstract

Papillary thyroid carcinoma often emerges within the middle-aged group as a painless, irregular thyroid mass, and it can be accompanied by other symptoms such as hoarseness and dysphagia. Cervical node metastasis typically involves the ipsilateral jugular chain and remains confined to cervical node levels III and IV in most of the patients. Here, we present a case highlighting the uncommon presentation of papillary thyroid carcinoma. A 63-year-old Malay man with no known medical issues initially exhibited a painless swelling on the right neck level V region. The swelling progressively grew in size and later extended toward the anterior and contralateral sides of the neck for a duration of a year. He reported a weight loss of 10 kg past a year and experienced consistent fatigue. Physical examination showed multiple firm swellings with regular borders over levels I to V bilaterally. The swellings did not move upon swallowing. Due to the absence of initial thyroid swelling and the presence of multiple cervical lymphadenopathies in his medical history, lymphoma was suspected. A referral to ENT was made for an excision biopsy, but the procedure was abandoned to prevent potential injury to important underlying structures such as blood vessels and nerves. An ultrasound-guided biopsy was subsequently performed over multiple neck regions. It resulted in papillary thyroid carcinoma instead of lymphoma. Despite a suggestive history of lymphoma, papillary thyroid carcinoma should be considered a potential differential for a multilobulated neck mass. Excision biopsy has the potential to upstage the tumor and worsen the patient's prognosis. Fine-needle aspiration for cytology should be prioritized when investigating any neck swelling.

## Introduction

Neck swelling is one of the most common complaints by patients in ENT practice. Any palpable masses between the inferior border of the mandible and till clavicle are usually defined as neck swelling. Neck mass can originate from one of these four regions thyroid, lymph node, salivary gland, and soft tissue swelling. It can be classified into benign or malignant tumors [1]. A thorough history taking, physical examination followed by fine needle aspiration cytology and imaging help the patient to get a proper diagnosis and initiate treatment promptly. As for papillary thyroid carcinoma, it often emerges within the middle-aged group as a painless, irregular thyroid mass, accompanied by hoarseness and dysphagia in 20% of cases [2]. Cervical node metastasis typically involves the ipsilateral jugular chain. It remains confined to cervical node levels III and IV in 73% of cases [3].

This article was previously presented as a poster at the Pahang Research Day 2023 on October 12, 2023.

## Case presentation

A 63-year-old Malay man, with no known medical illness, ex-smoker, and currently a pensioner, has presented painless right neck level V swelling that progressively increased and spread to the contralateral side of the neck for the past one year. It was associated with significant weight loss and lethargy. Otherwise, there were no obstructing symptoms such as hoarseness, dysphagia, shortness of breath, and stridor as well as aspiration symptoms. The patient denied any hypo/hyperthyroid symptoms.

**Figure 1:**
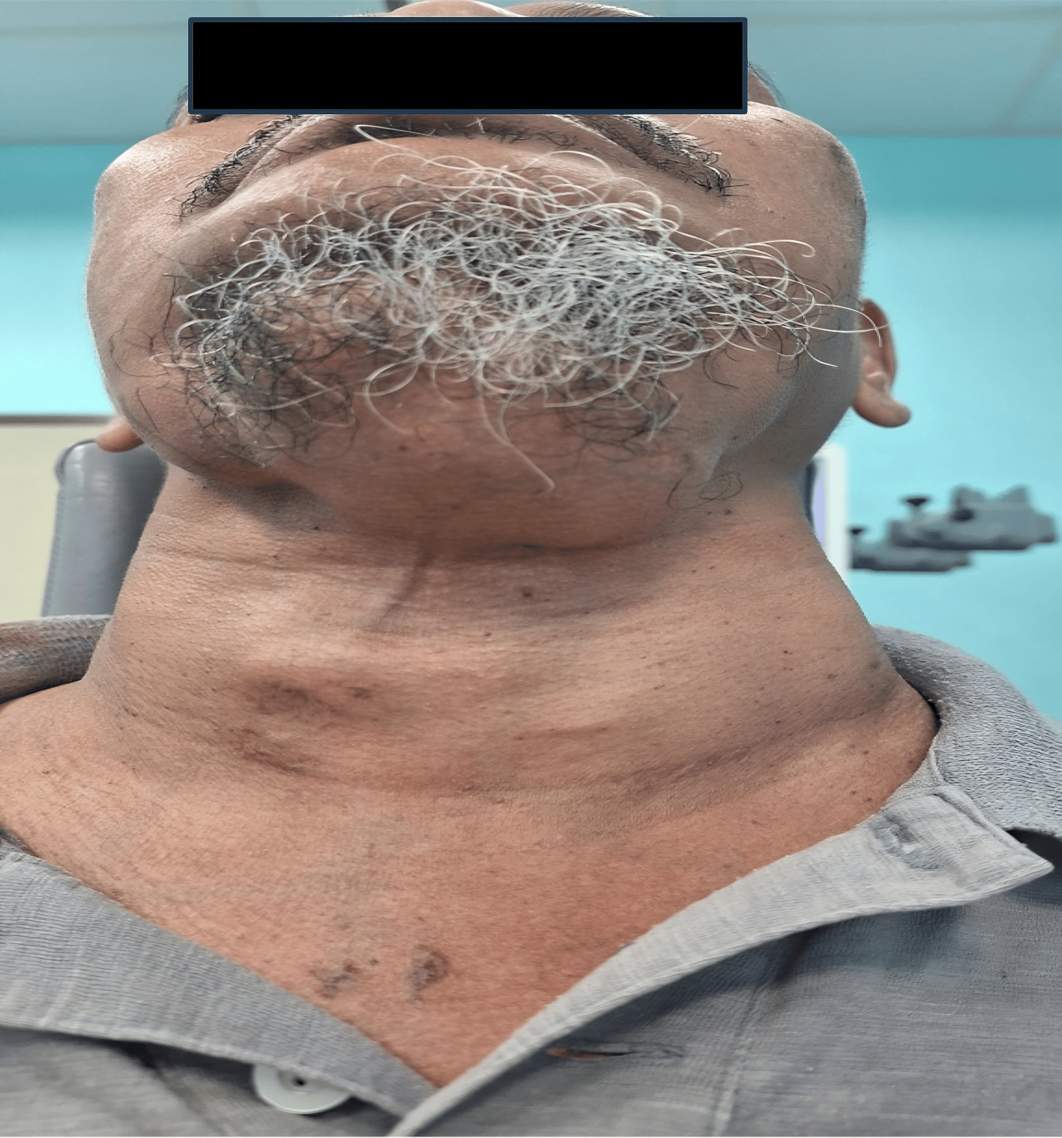
The patient presented with multiple neck swelling over level I-V with visible vessels over the anterior chest wall.

**Figure 2:**
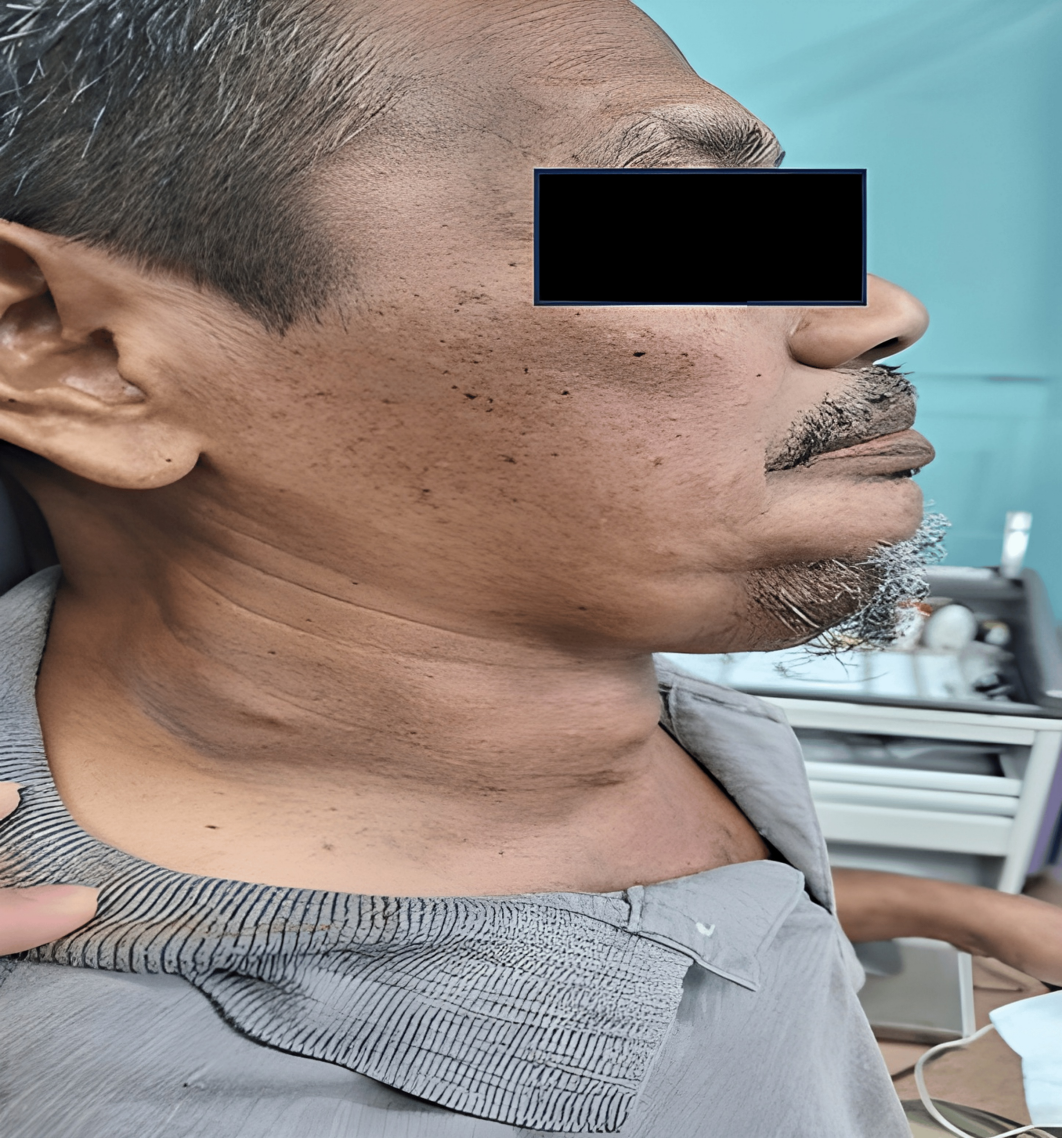
Lateral view of the patient neck showing multiple neck swelling.

Upon examination, there was multiple cervical swelling over level I-V, with the largest swelling over the submental region measuring 10x9cm. The swellings were firm, non-tender, and did not move with swallowing. Dilated vessels over the anterior chest wall were seen. Otherwise, no palpable axillary or inguinal lymph node (Figures 1, 2).

**Figure 3:**
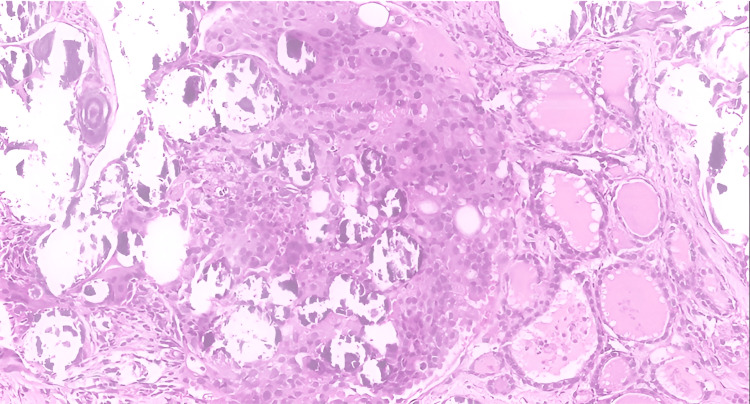
Papillary thyroid carcinoma is seen in the left thyroid gland. The malignant cells are arranged in solid and papillary architectures. Psammoma bodies are present (hematoxylin and eosin stain, х100 magnification).

**Figure 4:**
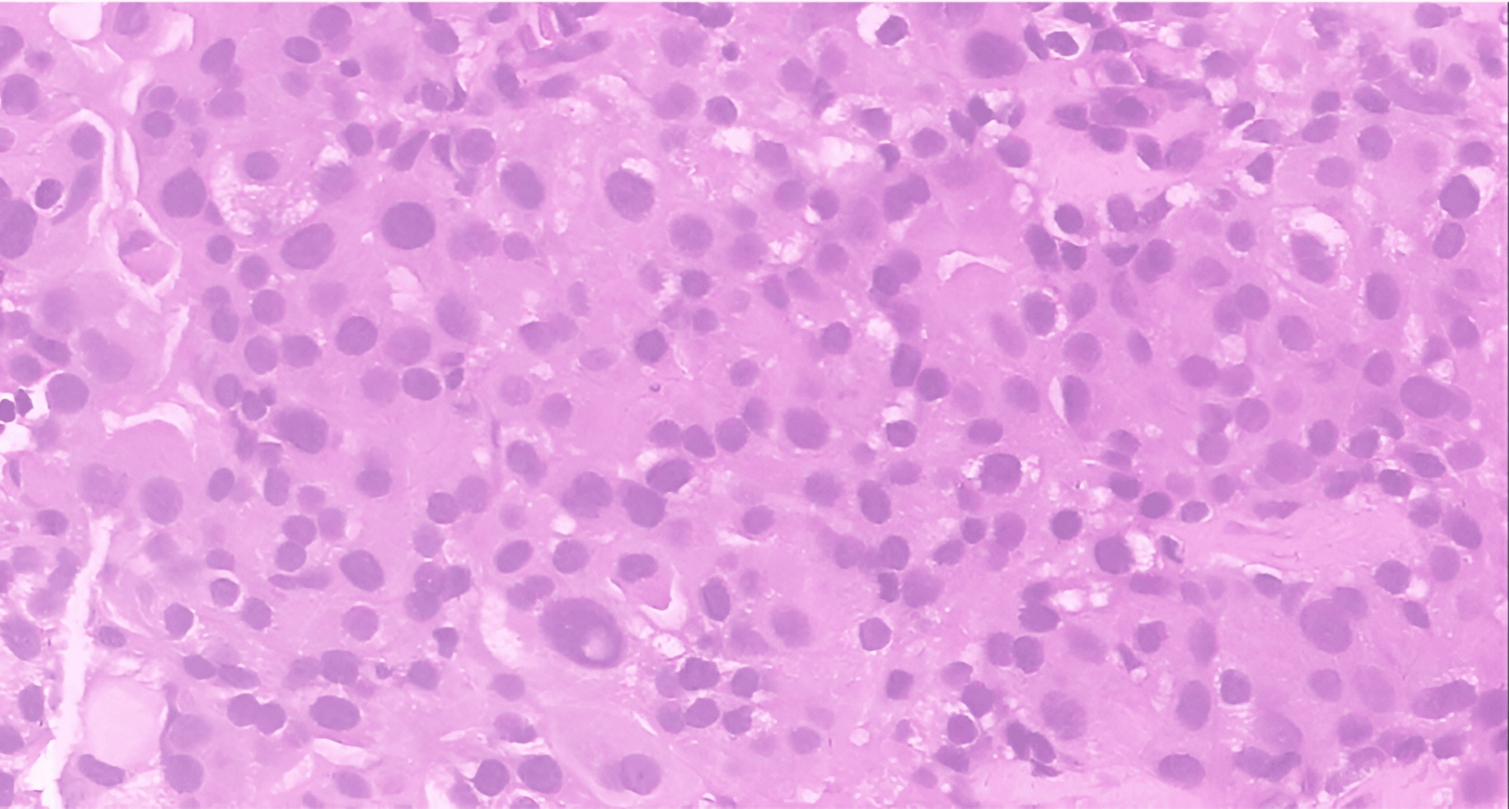
The histopathological specimen showed the papillary thyroid carcinoma exhibits moderate nuclear pleomorphism with nuclear grooving intranuclear pseudoinclusion (hematoxylin and eosin stain, х400 magnification).

Flexible nasopharygolaryngoscope showed airway patent with equal and mobile vocal cords. There was no medialization in his upper airway. Instead of an excision biopsy, ultrasound-guided biopsy was ordered and resulted in papillary thyroid carcinoma (Figures 3, 4).

**Figure 5:**
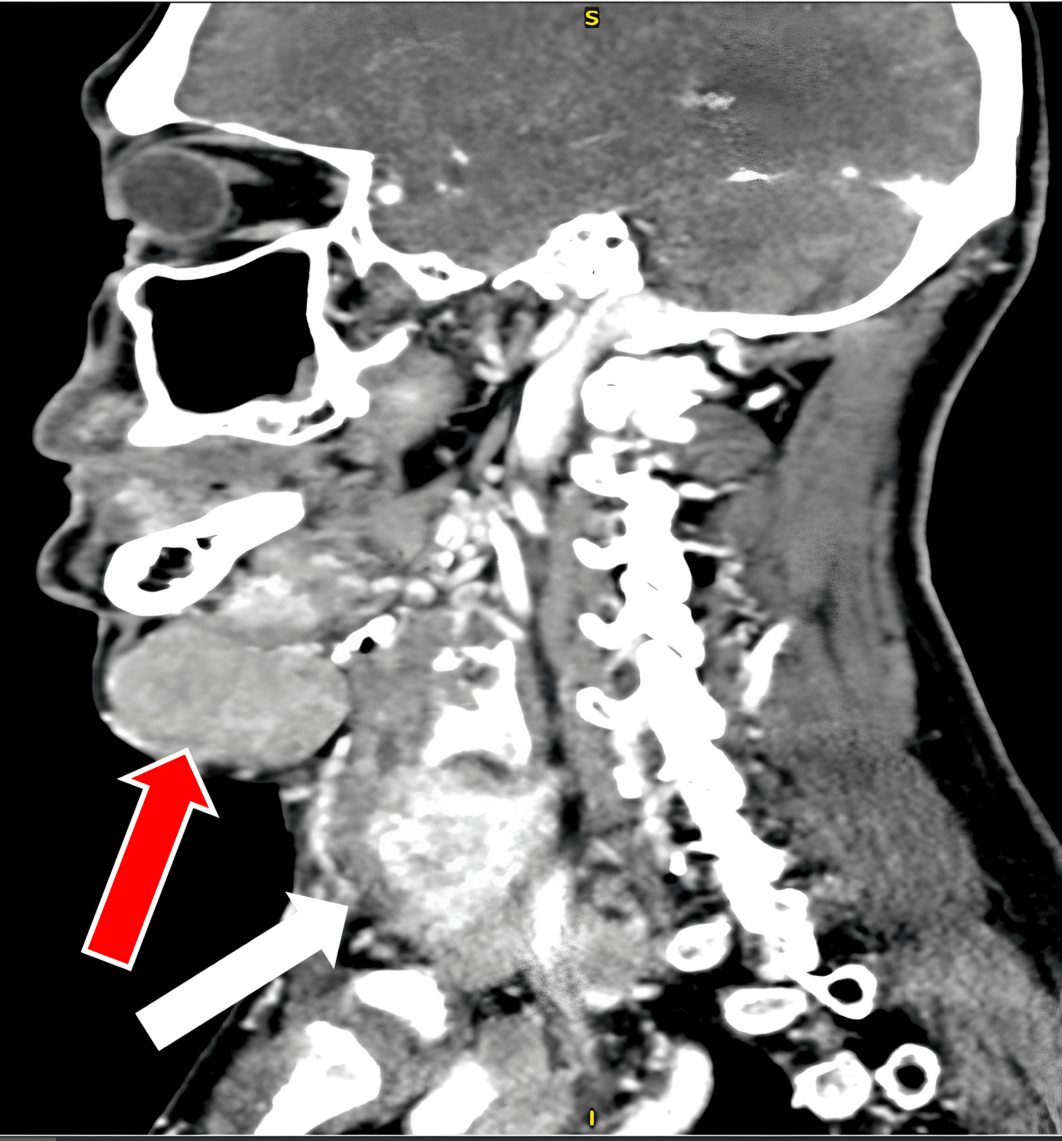
Sagittal view of the computed tomography neck and thorax for this patient. Findings showed bilateral cervical and mediastinal lymphadenopathies with lower cervical and central venous compression. Thyroid gland is heterogeneously enhanced and enlarged, and these lobes are displacing the trachea to the left. Bilateral lung nodules with manubrial lytic lesion are suspicious of metastasis. Red arrow points toward patient's submental cervical lymphadenopathy. White arrow points toward patient's thyroid region.

Contrasted computer tomography showed the thyroid gland is heterogeneously enhanced and enlarged and these lobes are displacing the trachea to the left. Multiple ill-defined hypodense nodules were seen in the right thyroid lobe. However, there was no retrosternal extension. Multiple enlarged enhanced lymph nodes are seen over bilateral cervical, supraclavicular, and superior mediastinal regions. The largest is at level Ia measures 4.5x6.8x4.2 cm and displaced the adjacent hyoid muscles superiorly. The largest mediastinal lymph node is located over the right paratracheal region measuring 3.2x3, 8x3.6cm. Enlarged mediastinal nodes are seen encasing the proximal aortic arch branches, brachiocephalic veins, bilateral internal jugular veins, and common carotid arteries. Pleural-based nodules are seen in the posterobasal segment of the right lower lobe and the posterior segment of the left upper lobe measures 0.7x0.9cm. Expansile lytic lesions are seen at the manubrium of the sternum (Figure 5). He was diagnosed with papillary thyroid carcinoma T4bN1bM1. The patient was subsequently referred to nuclear medicine for initiation of radioiodine ablation.

## Discussion

The most common presentation of papillary carcinoma of the thyroid is an asymptomatic (painless) mass at the level of the thyroid. In around 20% of cases, patients may present with dysphagia or hoarseness which likely indicates involvement of the recurrent laryngeal nerve and/or tracheal compression [2]. However, the patient did not present with any typical presentation of papillary thyroid symptoms, but the initial presentation was neck swelling over right level V. Besides, he presented with symptoms suggestive more toward lymphoma which are painless swelling, persistent fatigue, and B symptoms such as weight loss. B symptoms also known as symptoms of lymphoma, include night sweats, significant loss of weight, and high fever. That is why lymphoma was put as a provisional diagnosis.

Epidemiologically, women are three times more likely to have papillary thyroid carcinoma as compared to men. The mean age of presentation is usually 30 years old. The tumor size of papillary thyroid carcinoma is often between 1 and 3 cm only whereas in this case, the mass is extensive [2]. Three-quarters of the patients will be presented with cervical metastases. Lung and bone are the most common site distant metastases although distant metastases are rare [4].

It is rare to have papillary thyroid carcinoma manifested as lateral neck swelling without thyroid swelling as the first presentation. According to the literature review, only around 11% of thyroid malignancy is presented as a lateral cervical cyst. It can be either due to malignant transformation of ectopic thyroid tissue or metastatic spread to cervical lymph nodes from occult thyroid lesions [4,5].

Excision of lymph nodes should be refrained for non-lymphatic malignancy [6]. Despite this case being suspected as lymphoma, the latest systematic review in 2021, which consists of 47 studies of 7,268 patients, showed fine-needle aspiration for cytology provides high sensitivity (93%) and specificity (97%) in diagnostic tools for lymphoma [7].

Thyroidectomy is the mainstay of treatment for well-differentiated thyroid carcinomas. It is followed by radiotherapy with radioiodine ablation if the tumor is more than 4 cm or has significant lymph node involvement [2]. In this case, surgery could have been performed to reduce the bulk of the disease. However, the patient is not keen on any surgical intervention. He is only keen on oncological interventions such as radioiodine ablation.

## Conclusions

The initial presentation of lateral neck swelling can be one of the possible differentials for papillary thyroid carcinoma. Fine needle aspiration for cytology is a cost-effective tool to diagnose the cause of neck swelling. Ultrasound and CT guides will be required when the swelling is close to a vital structure or clinically not palpable lymph node. It would be followed with lymph node excision if fine needle aspiration for cytology is unable to provide a proper diagnosis. Although the initial provisional diagnosis is lymphoma, an excision biopsy without fine needle aspiration beforehand may lead to tumor seeding and upstaging of the papillary thyroid carcinoma.
